# Infant Emotional Mimicry of Strangers: Associations with Parent Emotional Mimicry, Parent-Infant Mutual Attention, and Parent Dispositional Affective Empathy

**DOI:** 10.3390/ijerph18020654

**Published:** 2021-01-14

**Authors:** Eliala A. Salvadori, Cristina Colonnesi, Heleen S. Vonk, Frans J. Oort, Evin Aktar

**Affiliations:** 1 Research Institute of Child Development and Education, University of Amsterdam, 1018 WS Amsterdam, The Netherlands; c.colonnesi@uva.nl (C.C.); vonk_heleen@hotmail.com (H.S.V.); F.J.Oort@uva.nl (F.J.O.); e.aktar@fsw.leidenuniv.nl (E.A.); 2 Research Priority Area Yield, University of Amsterdam, 1018 WS Amsterdam, The Netherlands; 3 Department of Clinical Psychology, Leiden University, 2333 AK Leiden, The Netherlands

**Keywords:** parent-infant interaction, parenting, infancy, emotional mimicry, affective empathy, mutual attention

## Abstract

Emotional mimicry, the tendency to automatically and spontaneously reproduce others’ facial expressions, characterizes human social interactions from infancy onwards. Yet, little is known about the factors modulating its development in the first year of life. This study investigated infant emotional mimicry and its association with parent emotional mimicry, parent-infant mutual attention, and parent dispositional affective empathy. One hundred and seventeen parent-infant dyads (51 six-month-olds, 66 twelve-month-olds) were observed during video presentation of strangers’ happy, sad, angry, and fearful faces. Infant and parent emotional mimicry (i.e., facial expressions valence-congruent to the video) and their mutual attention (i.e., simultaneous gaze at one another) were systematically coded second-by-second. Parent empathy was assessed via self-report. Path models indicated that infant mimicry of happy stimuli was positively and independently associated with parent mimicry and affective empathy, while infant mimicry of sad stimuli was related to longer parent-infant mutual attention. Findings provide new insights into infants’ and parents’ coordination of mimicry and attention during triadic contexts of interactions, endorsing the social-affiliative function of mimicry already present in infancy: emotional mimicry occurs as an automatic parent-infant shared behavior and early manifestation of empathy only when strangers’ emotional displays are positive, and thus perceived as affiliative.

## 1. Introduction

The tendency to automatically and spontaneously reproduce the emotional expressions observed in others, known as emotional mimicry, characterizes human social interactions across the life span [1,2]. Mimicry refers to the interpersonal coordination of movement between individuals both in timing and form [3], and has the crucial socially adaptive function of fostering similarity and affiliation [4]. Through mimicry, people communicate mutual understanding and feelings of warmth and liking, promoting positive interactions and social bonding [5,6,7]. Because synchronizing with others’ emotional facial expressions conveys the implicit message “I am like you” and hence “I feel you” [8], emotional mimicry has often been interpreted also in terms of basic, or low-level, empathy both in adults and infants, e.g., [9,10]. Although emotional mimicry represents one of the main forms of nonverbal communication, particularly for preverbal infants whose interpersonal exchanges consistently rely on emotional communication [11], little is known about the mechanisms modulating its development [12,13]. Identifying the factors that underpin individual differences in infant emotional mimicry seems paramount to foster our understanding of human communicative development. The present study investigated infant emotional mimicry of strangers’ facial expressions and its association with parent emotional mimicry, parent-infant mutual attention, and parent dispositional affective empathy.

### 1.1. Infant Emotional Mimicry

Preverbal social interactions predominantly rely upon facial expressions [14]; hence emotional mimicry represents one of the simplest, yet most essential, type of communication in infancy. Emotional processing abilities mature throughout the first year of life [15]. Already in the first months, infants are sensitive to expressive faces and are able to detect adults’ attentional and emotional behaviors. Over the first six months of life, infants significantly improve their visual perception abilities, from only discerning blurry features in the first few months to detecting relational information such as smiling and raised eye browns [16,17]. It is in between five and seven months that infants become able to distinguish and categorize the most basic emotions, including negative emotions such as anger and fear [18,19,20]. By the end of the first year of life, infants further learn to interpret others’ facial expressions to guide their behaviors in uncertain or novel situations [19,21]. Less is known, however, about the development of infants’ ability to reproduce the expressive behavior observed in others. While research has intensively questioned neonatal predisposition to imitate non-emotional facial expressions [22,23,24], only a handful of recent studies examined the early development of emotional mimicry [25].

The earliest evidence of emotional mimicry in infancy has been found at five months of age [26], but not earlier [9,13,27], in response to audio-visual laughing and crying emotional stimuli. At this age, infants can discriminate emotions from bimodal stimuli, while sensitivity to unimodal vocal and visual stimuli emerges at five and seven months, respectively [28]. Infant mimicry in response to positive facial expressions, such as happiness, has been systematically reported from age seven months onwards [9,13,27,29]. Results about mimicry of negative facial expressions have shown greater variability among investigations. For example, while prior research found seven-month-old infants to display emotional mimicry in response to sadness [13], fear [13,27], and anger [13], other studies did not support these findings [9,27,29]. Furthermore, like adults, infants were found to have a greater tendency to mimic positive (i.e., happy) than negative (e.g., sad, angry, fearful) emotions [29,30,31], due to their stronger affiliative nature. Notably, emotional mimicry in the first year of life seems to occur as a valence congruent response to another’s facial emotional display [13], and it is thus not necessarily an emotion-specific response. Taken together, emotional mimicry of certain basic facial expressions becomes part of the nonverbal communicative repertoire halfway through the first half-year of life, in between the fifth and the seventh month, and progressively develops in parallel with infants’ cognitive maturation and early socialization experiences.

Infant emotional mimicry is generally investigated with experimental cross-sectional studies that present the participants with (static or dynamic) videos of unfamiliar others’ facial expressions. In these emotion-eliciting situations, the most often used technique to assess the presence or absence of infant emotional mimicry has been facial electromyography (fEMG), which measures the presence (or absence) of subtle activity of the cheek and brow facial muscles, e.g., [9,27,29]. Recently, with an endeavor to account for the ecological validity of the gathered data, new behavioral perspectives were advanced as alternatives to capture emotional mimicry, such as microanalytic facial coding systems, e.g., [13]. These microanalytic methodologies rely on second-by-second offline coding of participants, so to capture micro changes in the facial-expressive behavior that would typically be imperceptible to the human eye. Given the growing focus that research has been devoting to the temporal aspects of adult mimicry, e.g., [32,33], the present study employs microanalysis to continuously measure infants’ and parents’ individual propensity to mimic rather than only assessing its presence (vs. absence). Continuous measures are particularly convenient when investigating the degrees of relation among experimental constructs, since they carry more information than dichotomous variables.

### 1.2. Parent Emotional Mimicry and Dispositional Empathy

Parents represent children’s most salient communication and social interaction models, especially in the first year of life. In fact, children learn and understand the world by observing, interpreting, and reproducing their parents’ behaviors during early socialization experiences [34]. It is not only infants who reproduce their parents’ facial expressions of emotions, but also parents repeatedly copy and mirror the expressive behaviors of their infants [35,36,37], generating a complex dynamic system of bidirectional mimicry processes [38,39]. Especially in the first six months of life, when infants spontaneously and involuntarily make use of positive or negative facial expressions, they enhance the same expression in their mothers [39,40,41,42]. It is thus reasonable to hypothesize that parents who tend to copy their infant’s expressive behaviors are more likely to have infants who also mimic their interaction partner more, whether this person is the parent [43] or an unfamiliar person [12,37]. Although the experience of being imitated is theorized to be crucial for children’s socio-cognitive development, empirical research on the topic remains scarce in the infancy period [44]. To our knowledge, no study has yet examined the concurrent relation between infant and parent emotional mimicry in triadic contexts of interactions, and therefore as response to a third partner (i.e., videos of strangers’ emotional expressions). This would be particularly relevant as infant and parent mimicry could be thus objectively discerned from one another.

Parents’ expressive behaviors, including mimicry, reflect individual dispositions (or traits) to communicate and interact with others. A personal characteristic strictly interrelated with emotional mimicry is empathy [45,46], and particularly affective (or emotional) empathy (i.e., capacity to experience and share the emotional state expressed by others; [47]). Prior research has shown that parents who experience high levels of affective empathy (i.e., feelings of care and concern for less fortunate individuals in distress) tend to have children who also exhibit more empathic behaviors toward others in need, e.g., [48,49]. No research, however, has investigated the relation between parental empathic dispositions and children’s emotional mimicry behaviors during infancy.

### 1.3. Emotional Mimicry and Mutual Attention

During early parent-infant social interactions, episodes of emotional mimicry are often embodied into moments of mutual attention, during which the interaction partners simultaneously look at each other [50]. These moments of gaze synchrony are considered one of the earliest and most salient types of automatic mimicry [51]. They are functional for both interaction partners to learn how to communicate by temporally coordinating their nonverbal behaviors not only within the dyad but also toward third stimuli (e.g., objects, events, people). For example, around the end of the first year of life, infants learn how to make use of their parents’ attentional and emotional responses to interpret ambiguous situations and regulate their emotions to a comfortable level, an ability known as social referencing, e.g., [21,52]. Parents’ engagement in mutual attention with their infant, on the other hand, has often been interpreted in terms of sensitivity and responsiveness to the infants’ feelings and needs [53].

The idea that mutual attention and emotional mimicry are closely related is compelling because both emerge and manifest as synchronous and affiliation-driven behaviors. Prior adult and infant studies indicated that when participants are passively observing a model’s non-emotional facial expressions (i.e., eyebrow and mouth actions), the model’s direct (vs. indirect) eye contact enhances participants’ mimicry [54,55]. Examinations into the association between mutual attention and emotional mimicry in triadic contexts of interaction is of great interest since the attention shared between the interaction partners might foster the dyad’s understanding of the social situation, and, in turn, their emotional mimicry.

### 1.4. The Present Study

The present study investigated the associations among infant and parent emotional mimicry of strangers, parent-infant mutual attention, and parent dispositional affective empathy. Infants and their parents were presented with dynamic videos of unfamiliar adults’ happy, sad, angry, and fearful facial expressions. The inclusion of multiple negative emotional stimuli in the study design enabled us to examine participants’ behavioral responses as a function of the specific emotion rather than solely of its (negative) valence. In order to detect possible developmental differences in the first year of life, we selected infants from two age groups, namely at six and twelve months, a period that characterizes not only the development of the ability to discriminate among various negative facial expressions [56] but also the onset and peak of triadic communication [57].

We hypothesized that: (a) parent dispositional affective empathy directly relates to the parent emotional mimicry; (b) parent emotional mimicry directly relates to infant emotional mimicry; (c) parent dispositional affective empathy and infant emotional mimicry are directly and indirectly related, through parent emotional mimicry; (d) parent-infant mutual attention and infant emotional mimicry are directly and indirectly related, through parent emotional mimicry.

## 2. Materials and Methods

### 2.1. Participants

Participants were six- and twelve-month-olds and one of their parent (mother or father) living in Amsterdam and surrounding areas. Parents were recruited through an information letter distributed by the municipality of Amsterdam. Families were part of a larger eye-tracking research project on early emotion processing in six-, twelve-, and eighteen-month-olds. We included all participants for whom: (a) infants were in their first year of life (six and twelve months); (b) infant and parent were visible to one another during the experimental procedure (i.e., parent sitting next to the infant and in front of the video); (c) absence of fussiness. The final sample consisted of 117 infant-parent dyads: 51 six-month-old infants (24 girls; *M_age_* = 6.17 months; *SD_age_* = 0.48; range = 5–7 months) and 66 twelve-month-old infants (41 girls; *M_age_* = 12.07 months, *SD_age_ =* 0.62; range = 10.66–13 months) and one of their parents (35 and 50 mothers, in the six- and twelve-month age groups, respectively; *M_age_* = 34 years, *SD_age_* = 4.69, range *=* 26–62 years). The majority of parents participating in the study (68%) were of Dutch origin, and the remainder included Italian, German, Romanian, British, and Polish nationalities.

### 2.2. Research Design

Participants were tested at two-time points. First, parents were invited to complete an online questionnaire booklet consisting of socio-demographic and empathy-related questions. Second, infants were invited to the baby laboratory of the University of Amsterdam to take part in the interactive session with one of their parents. Written informed consent was obtained from all parents prior to starting the experiment. Data were already collected at the time of the design of the study.

### 2.3. Questionnaire: Parent Dispositional Empathy

Parent affective empathy was assessed with the sub-scale “empathic concern” of the Dutch version of the Interpersonal Reactivity Index (IRI; [58,59]). Empathic concern represents the other-oriented dimension of affective empathy and measures the individual tendency to experience feelings of sympathy or concern for unfortunate others on a 7-point Likert scale (e.g., item, “I often have tender, concerned feelings for people less fortunate than me”). Internal consistency of the empathic concern scale was α = 0.68, similar to those reported with the Dutch population (α = 0.73; [59]).

### 2.4. Interactive Task: Procedure and Emotional Stimuli

**Figure 1:**
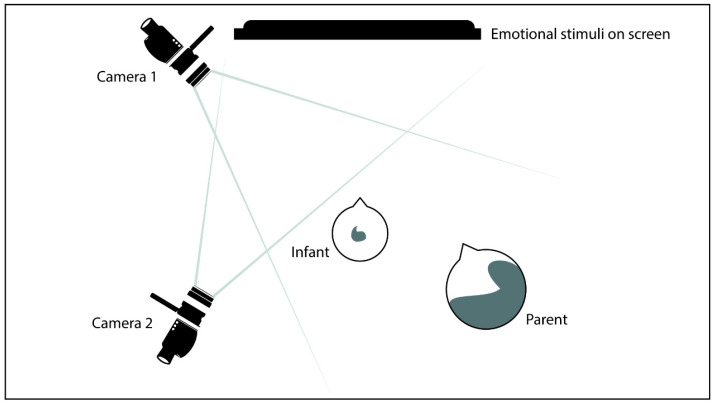
Triadic setting.

Before starting the experiment, parents were invited to place their infant in a car seat mounted on a table in front of a computer screen (1280 × 1024 pixels) and to seat themselves on the chair located on their infant’s right side, so to create a triadic setting (see Figure 1). Because the main interest of this study was on the natural unfolding of the social interaction, and thus on participants’ automatic and spontaneous behavioral responses, parents were not explicitly told to watch the video displayed on the screen. Instead, they were simply instructed to behave as they would normally do and not to intervene unless the child sought for their attention. In the eventuality of distress or fussiness, parents were encouraged to comfort their babies by soothing them. Throughout the experimental paradigm, a twin camcorder (Panasonic HC-W570) recorded the triadic contexts on video: the main camera recorded the parent’s and the infant’s face and upper body, capturing them in the main window; the second camera recorded the emotional stimuli presented on the screen, capturing them on a small window.

**Figure 2:**
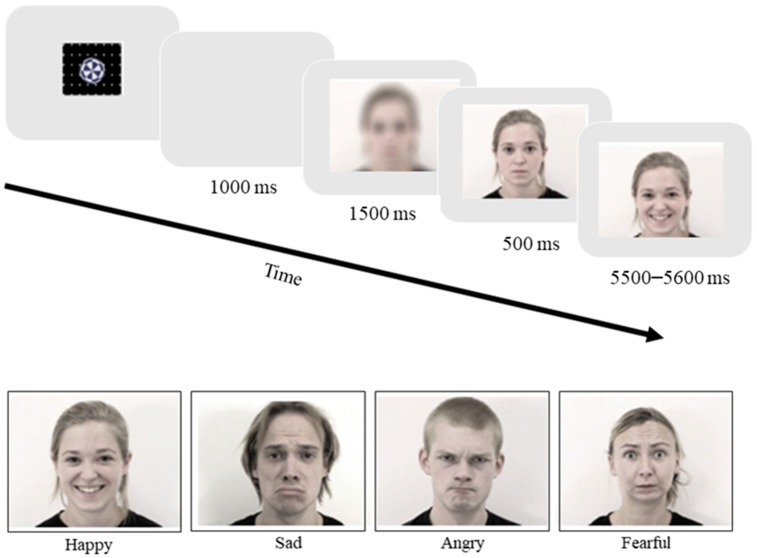
Time flow of the trials and examples of the emotional stimuli.

Emotional stimuli were employed from the Amsterdam Dynamic Facial Expression Set (ADFES), which consists in validated and standardized dynamic videos featuring facial expressions of emotions [60]. Facial expressions are based upon prototypes of the “basic emotions” as described in the Facial Action Coding System (FACS) Investigator’s Guide [61]. In order to prevent gender biases, both female and male adults were included as stimuli. Participants were presented with eight blocks, two for each of the four models. Each block consisted of 5 trials: neutral, happy, sad, angry, and fearful facial expressions. Blocks always started with the neutral trial, intended as familiarization to the model’s face, and proceeded with presentation of the other trials in a randomized order. Each trial started with 500 ms of the attention-getter video (repeated if the child was not attentive), followed by 1000 ms of blank screen and 1500 ms of blurred face. The model’s facial expression became then clearly visible, and stayed neutrally static for 500 ms, followed by the dynamic unfolding of the emotional expression that reached the apex of the expressive behavior in 500 ms, and stayed at the apex for 5000 ms. Figure 2 illustrates the time flow of the trials and examples of emotional stimuli.

#### 2.4.1. Behavioral Coding System

Observational data were analyzed at the micro-level (coding with units of 1 s or less, up to an accuracy of 1/25th of a second) using the computer software The Observer XT 12.5 [62]. The first four blocks, corresponding to one block for each of the four models, were systematically coded. Coding was restricted to the time intervals when there was a facial expression on the screen (i.e., 6000 ms). The coding of the infant and the parent were performed independently by distinct coders in separate coding sessions. The infant coder was trained to code the emotional stimulus as well. A total of six coders, three for each age group of the infant (i.e., six and twelve months), participated in the project.

Colonnesi et al. [11]’s coding system for emotional communication was adopted. Accordingly, facial expressions and gaze were coded as state events (i.e., duration in seconds) into specific mutually exclusive categories. Facial expressions were identified as positive when involving smiles and raising corners of the lips, and as negative when involving frowns or lowered-lip corners. Neutral facial expressions were coded when neither a positive nor a negative facial expression was displayed, as no muscle movement was visible, or the visible muscle movements were not indicative of an emotion. Gaze direction was coded as to the stimulus, the screen, the interaction partner, or elsewhere in case of neither of the prior ones. An additional coding category was included to filter the observational data on the basis of the emotional stimuli displayed by the videos on the screen, so to classify them as neutral, happy, sad, angry, or fearful.

A total of 14 videos for each age group of infants, corresponding to 26% and 21% of the recordings of the two groups, respectively, were randomly selected and double coded to perform inter-rater reliability. The mean average Cohen *k’s* yielded from the reliability coding of participants’ behaviors in the six- and twelve-month age groups were as follows: infant facial expressions 0.90 and 0.93; parent facial expressions 0.83 and 0.93; infant gaze 0.95 and 0.93; and parent gaze 0.97 and 0.96. These values are comparable to those of other studies that used similar microanalytic methods, e.g., [11], and are considered exceptionally good as the reliability was based on agreements not only in scored behavior, but also in time of coding.

#### 2.4.2. Data Preparation

**Figure 3:**
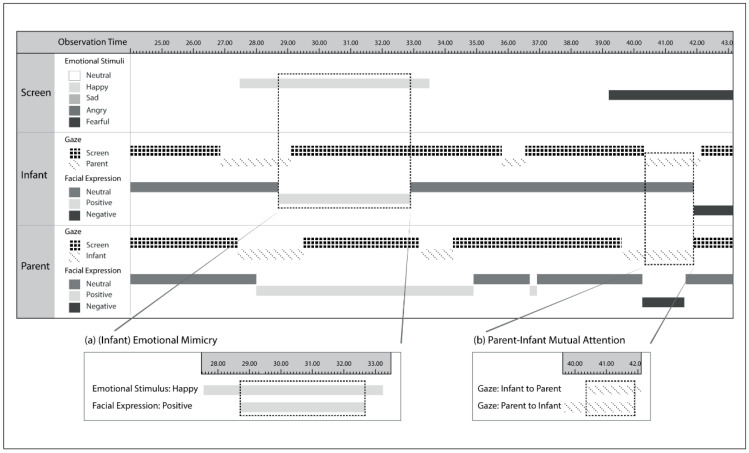
Data visualization examples. (**a**) Emotional mimicry, operationalized as temporal co-occurrence) of the infant’s negative facial expression to the sad emotional stimulus. (**b**) Parent-infant mutual visual attention, operationalized as temporal co-occurrence of parent’s and infant’s gaze toward each other.

Emotional mimicry and mutual attention scores were computed separately for each emotion. Emotional mimicry was quantified as the time (duration in seconds) spent in displaying a valence-congruent facial expression of the emotional stimuli. In other words, emotional mimicry was identified when participants displayed positive facial expressions (i.e., smiling) in response to the happy stimuli, and negative facial expressions (i.e., frowning/scowling) in response to the sad, angry, or fearful stimuli. Parent-infant mutual attention was quantified as the time (duration in seconds) spent by the infant and the parent simultaneously looking at one another. Figure 3 illustrates an example of data visualization.

Finally, emotional mimicry and mutual attention were turned into percentage scores, based on the total length of the specific-emotion stimulus presentations (e.g., total time spent displaying positive facial expressions during the happy stimuli presentations/total time of the happy stimuli presentations * 100).

### 2.5. Statistical Approach

Missing data inspections revealed that six parents (5% of the total sample) did not complete the IRI questionnaire. As data were missing completely at random, Little’s Missing Completely at Random (MCAR) test χ 2 (6) = 8.55, *p* = 0.200, we used the Expectation Maximization (EM) approach to estimate the variance-covariance matrix [63]. Normality of the data was examined by checking skewness and kurtosis and dividing these statistics by their standard errors: when exceeding one standard deviation ±1.96 the variable was assumed to deviate from normality significantly. We found a moderate degree of positive skewness for the percentage scores, which improved after dealing with univariate outliers (i.e., *z*-scores exceeding ±3.29 were winsorized by assigning them with scores one unit larger than the next most extreme score) [64]. Statistical log-transformations were also computed, and analyses were run on both original and transformed data. Since similar results were yielded, analyses with the original variables are presented.

**Figure 4:**
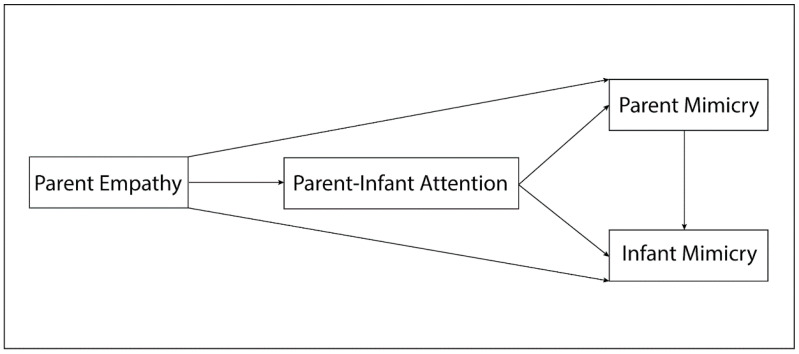
Conceptual path model linking infant emotional mimicry, parent emotional mimicry, parent-infant mutual attention, and parent dispositional empathy.

Bivariate Pearson’s correlations were performed to test the intra-constructs relations across the presentation of happy, sad, angry, and fearful stimuli, separately for measures of parent and infant emotional mimicry, and parent-infant mutual attention. The hypothesized direct and indirect relations among the experimental constructs were simultaneously tested with path analyses, a subtype of structural equation modeling (SEM). Figure 4 illustrates the conceptual model. Four recursive path models were constructed, separately for the happy, sad, angry, and fearful stimuli. Path coefficients, which indicate the strength and the directions of the relations between the constructs, were interpreted in standardized metric and considered significant at *α* = 0.05 level. Only significant paths are reported in text, and since models were fully saturated (i.e., just identified), no fit index is presented. Path analyses were performed in R (version 3.5.1) with the Lavaan package [65].

### 2.6. Preliminary Analyses

Prior to the main analyses, we performed independent-sample *t*-tests on each of the experimental variables to examine the effect of infants’ age. As no significant differences between six- and twelve-month-olds were revealed, the two age groups were merged together for the final analyses. Table 1 displays the mean, standard deviations, and ranges of the experimental variables.

The correlations within the experimental constructs are presented in Table 2. Significant positive correlations were found between parent mimicry of happy, sad, angry, and fearful stimuli. Differently, significant negative correlations were found between infant mimicry of happy stimuli and angry and fearful stimuli. In other words, parents who mimic others’ positive facial expressions longer are also those who mimic others’ negative facial expressions longer, while infants who mimic others’ positive facial expressions longer are those who mimic others’ negative facial expressions shorter. Inter-relations among the measures of participants’ emotional mimicry of negative stimuli (i.e., sad, angry, and fearful stimuli) were significantly positive in parents as well as in infants, meaning that the tendency to mimic a negative emotion is similar across discrete negative emotions. Moreover, measures of parent-infant mutual attention were all positively inter-related. Accordingly, the parent-infant dyads who share longer mutual attention during the presentation of a certain emotional stimuli are also those doing so during presentation of any other emotional face.

A preliminary MANOVA was conducted to investigate the role of the distinct emotion on the experimental variables. Overall, a significant result was found, *F*(9, 1124) = 30.85, *p* < 0.001, η2 = 0.16. Post hoc tests pointed out that: (I) both infants and parents mimic positive (vs. negative) stimuli longer (*p_s_* < 0.001); (II) both infants and parents do not show significant differences in the duration of their emotional mimicry of negative stimuli (i.e., sad, angry, and fearful stimuli); (III) there are no significant differences in the duration of parent-infant mutual visual attention during presentation of positive and negative stimuli.

## 3. Results

**Figure 5:**
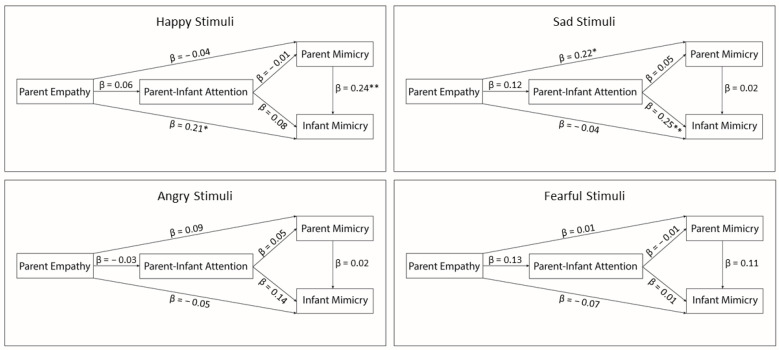
Path coefficients, which indicate the strength and the directions of the relations between the experimental constructs, are reported in standardized metric, and were considered significant at the α = 0.05 level. * *p* < 0.05; ** *p* < 0.01.

Figure 5 illustrates the direct effect coefficients estimated for each of the path models. When happy stimuli were presented, infant emotional mimicry was positively associated with parent emotional mimicry and affective empathy, *β* = 0.24, *SE* = 0.07, *p* = 0.007, and *β* = 0.21, *SE* = 0.02, *p* = 0.017, respectively. Hence, the infants who mimic the happy stimuli longer have parents who both mimic longer and score higher levels of affective empathy. No relation was found between parent emotional mimicry and their affective empathy nor between parent-infant mutual attention and infant emotional mimicry.

When sad stimuli were presented, infant emotional mimicry was positively associated with parent-infant mutual attention, *β* = 0.25, *SE* = 0.22, *p* = 0.005, indicating that infants who mimic the sad stimuli longer are those who share longer attention with their parents. In addition, parent mimicry of the sad stimuli significantly related to their levels of affective empathy, *β* = 0.23, *SE* = 0.01, *p* = 0.016, showing that parents with higher levels of dispositional affective empathy are those who mimic longer strangers’ facial expressions of sadness. Neither parent-infant mutual attention nor infant emotional mimicry was related to parent emotional mimicry or their affective empathy.

When angry or fearful stimuli were presented, no significant relation among the experimental constructs was found. Additionally, no support was provided to the hypothesis that parent dispositional empathy and emotional mimicry are related to infant emotional mimicry through parent-infant mutual attention since no indirect effect yielded to statistical significance.

Additional exploratory analyses to examine potential effects of the gender of the parent participating in the study were performed, by modeling it in the path analyses as a control variable. Results revealed that mothers score significantly higher levels of affective empathy than fathers, an effect found to be stable across all the path models, *β* = −0.27, *SE* = 0.16, *p* = 0.003. Furthermore, compared to fathers, mothers mimic significantly longer the fearful stimuli, *β* = −0.18, *SE* = 0.02, *p* = 0.049, and share significantly longer attention with their infant during presentation of sad stimuli, *β* = −0.21, *SE* = 0.01, *p* = 0.022. When modeling infant gender or parent gender * infant gender, no significant effect was found.

## 4. Discussion

The present study examined the associations among infant and parent emotional mimicry of strangers, parent-infant mutual attention, and parent dispositional affective empathy. The main hypotheses concerned the extent to which parent dispositional affective empathy and their mimicry behaviors affect the infant mimicry during a triadic situation in which they are presented with emotional stimuli (i.e., videos of unfamiliar adults’ facial expressions of emotion). Results indicated that infant emotional mimicry of happy stimuli was positively and independently associated with parent mimicry and affective empathy. Infant emotional mimicry of sad stimuli was related to longer parent-infant mutual attention but to none of the parental dimensions. Parent dispositional affective empathy was positively related to their own emotional mimicry of sad stimuli. Infant emotional mimicry of angry and fearful stimuli were associated with none of the parent or parent-infant experimental constructs. These findings support previous theoretical and empirical evidence on the development of empathy and also provide new insights into the infants’ and parents’ coordination of mimicry and attention during triadic contexts of interaction.

### 4.1. Parent Dispositional Affective Empathy and Parent Emotional Mimicry

Parent dispositional affective empathy was positively related to their emotional mimicry of strangers’ sad facial expressions. In other words, parents who reported to have stronger empathic traits were more predisposed to engage in episodes of emotional mimicry when observing someone’s sadness. These findings endorse the association between self-reported measures of empathy and observed mimicry behaviors [45,46], further supporting the assumption of mimicry as a mechanism to regulate affiliation. Among the basic emotions, sadness is the only one that conveys requests of support, help, and comfort [66,67]. As such, sad displays result highly affiliative and elicit reactions of empathy and compassion toward the expresser [48,68]. Mimicking other people’s sadness signals an understanding of their suffering and willingness to help [31]. Hence, it is likely that parent affective empathy, measured as means of empathic concern for another person in distress, was particularly activated during observation of sad stimuli [69]. Furthermore, as the parent shared the view of the stranger’s sadness together with their infant, responses to these sad stimuli might also reflect the parent’s sensitivity and responsiveness in such triadic contexts of interactions.

In addition, no relation was found between parent dispositional empathy and their observed emotional mimicry of happy, angry, and fearful stimuli. A possible explanation regards methodological reasons: the items of the empathic concern sub-scale employed from the IRI questionnaire to measure affective empathy are mainly geared toward assessing reactions to others’ distress [69], as for example “I often have tender, concerned feelings for people less fortunate than me”, or in its inverse formulation “Other people’s misfortunes do not usually disturb me a great deal” [58]. This might have limited the generalization of parental affective empathy to situations that specifically elicit concern and compassion for others’ distress, such as sadness. Anger and fear, on the other hand, are emotions that do not necessarily signal others’ misfortune. This interpretation could also explicate why parent empathy and their emotional mimicry were found to be negatively correlated (although not significantly) in the case of happy stimuli. Happiness reflects the opposite of being concerned for someone who is less fortunate or in trouble.

### 4.2. Parent Emotional Mimicry, Parent Dispositional Affective Empathy, and Infant Emotional Mimicry

As expected, infants who displayed longer emotional mimicry of happy stimuli had parents who also displayed longer mimicry. This result corroborates and extends recent findings on the development of emotional and non-emotional facial mimicry, which was found supported by early parental imitation [12,37]. Prior research has shown that levels of received and produced facial mimicry co-vary during infants’ early interactions with their mothers [39,70]. Western infants spend approximately 65% of their awake time in face-to-face interactions with caregivers [42], and matching behaviors between partners occur once every minute, mostly as the results of caregiver imitation of the infant [41]. Furthermore, observational studies systematically indicated that young infants instantly tune in to changes in their parents’ expressions of emotion, mirroring them [71]. Thus, infants are more positive when parents express more positive affect [72]. These early face-to-face interactions are suggested to form the basis of emotional mimicry [73]. Presumably, infants learn to recognize and reproduce another’s facial expressions of emotions through both interaction with their parents [74] and observation of parents’ emotional expressions to novelty [75]. In fact, the nonverbal signals that children observe being directed toward unfamiliar individuals are known to influence their attitudes and behavior toward those individuals, e.g., [76,77]. This type of observational social learning is essential for children to learn about others in their social world [78]. Our findings extend previous knowledge, suggesting that already in the end of the first half-year of life infants and their parents automatically and spontaneously share positive affect when in triadic contexts of interactions. Crucially, they do so only when the emotional stimuli are happy, and thus perceived as affiliative. In fact, mimicry is most likely to occur when there is a shared understanding and thus a shared reaction to a stimulus [31,79].

Infant emotional mimicry of happy stimuli was also positively, but independently from parent mimicry, related to the parent level of dispositional affective empathy. Our findings endorse the association between parent dispositions and children’s socio-emotional development [49,80] and extend previous evidence to the infancy period, suggesting that parents who score higher in affective empathy tend to have infants who exhibit more empathy-related behaviors, such as emotional mimicry. Nevertheless, the hypothesis of indirect pathways from parent dispositional affective empathy to infant mimicry via parent mimicry was not supported. Possibly, infant emotional mimicry of happy stimuli is independently related to a similar mimicry of parents because of interactive factors (i.e., shared positive emotionality) and to parental empathic dispositions through more hereditable mechanisms (e.g., shared parent-child characteristics). This interpretation is in line with prior research examining the genetic contributions in the intergenerational transmission of empathy from parent to child, which proposes that affective empathy might indeed have substantial hereditability estimates and may thus occur in a rather direct manner (i.e., through genes), while socialization processes may be more related to the transmission of cognitive empathy [81,82].

The fact that associations between infant emotional mimicry and both parent mimicry and empathy were found during presentation of positive, but not negative, stimuli is intriguing. In line with the contextualized view of emotional mimicry in adults [79], mimicry is sensitive to contextual cues, such as the valence of the nonverbal emotional display. As it serves a social-affiliative function, emotional mimicry is likely to occur as a parent-infant dyad-shared behavior and early manifestation of empathy only when strangers’ emotional displays are positive, and thus the social context is perceived as affiliative. Happy faces can be easily interpreted as they do not create confusion or ambiguity and do not require any regulation of emotional distress [83]. Because of their clear communicative intent, positive (vs. negative) emotions are more frequently mimicked [2,30], tend to be mimicked faster [84], and regardless of the situational context [85] or the relationship with the expresser [30]. Negative emotions, on the other hand, are known to demand more advanced levels of cognitive information processing [79], and might require appraisal [86], especially if the observer is an infant who has been predominantly exposed to parents’ happy facial expressions [74]. The implication of additional cognitively loaded interpretations might be what influenced the parent-infant dyad’s mimicry responses to negative emotions, making them not as automatic and spontaneous as they were found to be for positive emotional mimicry. This type of negativity bias has been indeed priorly observed among infants and young children, e.g., [87,88].

The lack of association between infant mimicry of negative expressions and parent mimicry and empathy could also be explained in terms of interactive factors and positivity bias, as infants in the present study may have shown greater mimicry in response to facial expressions that are most familiar and most relevant in their daily social interactions such as happiness [15,89,90]. When a child is displaying a negative facial expression, even if this is an episode of mimicry, the parent might be more likely to respond with happy expressions, rather than mimicry, so to regulate the infant’s emotion [91] and communicate them that the situation perceived as sad or distressed is actually safe [92]. This might be particularly compelling for infants of parents with high affective empathy. As parent affective empathy was measured as empathic concern to the misfortune of others, it plausibly captured parental sensitivity, compassion, and tenderness as well. It seems thus reasonable that the parents who scored high in affective empathy in the present study were also those behaving more sensitively to their children’s negative emotions, responding to them with happy expressions, rather than mimicry, in order to regulate infants’ possible negative affectivity and calm them down [92]. Happy facial expressions might, therefore, represent very familiar and recognizable emotional stimuli for children of empathic parents, and as such these emotions of happiness are automatically processed and mimicked.

### 4.3. Parent-Infant Mutual Attention and Infant Emotional Mimicry

A particularly interesting interactive dynamic was elicited during the presentation of sad stimuli: the infants who displayed longer emotional mimicry were those who engaged longer in mutual attention with their parents. Sadness is probably the most familiar negative emotion to younger infants, as commonly reinforced by mothers in social interactions between the third month and the end of the sixth month of life [36]. Yet, observing strangers’ sadness without being provided with any contextual information to interpret such negatively valenced emotion might have elicited confusion and disorientation in the infant. Under uncertainty, affiliation with others who share the same situation may result in the best way of evaluating the intensity, nature, or appropriateness of one’s emotional state [93]. In order to regulate appraisals of uncertainty, people tend to socially compare their own emotional responses to those of similar others [94] and to seek for social information that can help them to reduce these negative and uncertain feelings. Utilizing others’ emotional communication as a social reference has socially adaptive functions [52]. By the end of the first year, it is common for infants to use parents’ emotional and attentional cues to appraise the situation and learn about the social context [19,21], especially when in an uncertain or ambiguous situation [92]. For example, infants tend to engage this strategy to regulate themselves and to infer how to (emotionally and behaviorally) respond to both familiar and novel stimuli [95]. Conjointly, parents do visually check on their infant to ensure there is no overwhelm or worry. As young infants’ self-regulatory capacities are not completely matured [96], sharing visual attention with their infants could have represented for parents the context to regulate infants’ socio-emotional state by providing them with emotional guidance. These early socialization contexts are fundamental in shaping the infant’s future strategy for expressing and regulating emotions [79].

The fact that a significant association between parent-infant mutual attention and infant emotional mimicry occurred only with sad stimuli, but not with other negatively valenced facial expressions, is striking. In line with the functionalist perspective, each negative emotion elicits distinct responses from the interaction partner, as it has a distinct adaptive social function [97,98]. Anger and fear, for example, are emotions that represent threat-related stimuli [99], and are thus supposed to particularly require infants’ appraisal of the ambiguous environment. It is thus surprising that infants’ responses to angry and fearful stimuli did not relate to the dimensions of parent-infant mutual attention, nor to parent emotional mimicry. One possible reason for this finding is that anger and fear, differently from sadness, are emotions that particularly communicate self-oriented (vs. other-oriented) prompts and might be thus perceived as less affiliative [66,68]. Indeed, whereas sad expressions convey requests of support, help, and comfort, and therefore tend to elicit affiliation and interaction, angry and fearful expressions are suggested to have distancing functions and to trigger withdrawn behaviors in the observer, communicating the cessation of the activity [66,68,100].

### 4.4. Research Implications

Empirical research on infant emotional mimicry has important clinical implications. One socially adaptive function of emotional mimicry is the understanding of others’ thoughts and emotions [101]. Human natural tendency to automatically mimic the facial expressions of their interaction partner is believed to be delayed or reduced in individuals with poor socio-emotional functioning, such as people with autism spectrum disorder [102,103,104]. Their impairments in social communication, such as lack of visual attention to social stimuli and atypicalities in the (re)production of facial expressions [105], have been suggested to reinforce each other throughout development, leading to further deficits in social cognition [106], which are observable as early as within the first year of life [107,108]. Early and intensive behavioral interventions that provide interaction and communication stimulation might thus have profound and beneficial impacts on children identified as having difficulties in social communication [109], including emotional mimicry.

As well as considering the importance of screenings and early interventions, research toward a better understanding of the specific parental factors conditioning children’s behaviors as early as in the first year of life is also paramount. Parents’ own emotion-processing and regulation impact their children’s emotional development [110]. For example, there is evidence that low parental dispositional empathy is associated with an increased abusive and neglectful parenting [111]. Another line of research has also demonstrated that children of depressed or anxious parents are at increased risk of being diagnosed with such disorders themselves [112]. In fact, parents’ depression and anxiety is associated with less positive expressive behavior during interactions with their children [75], including decreased imitation [113], which results in lower levels of imitation in their children as well [113]. Together, these findings implicate that similarities between the parent and the infant are due to biological predispositions and genetic influences as well as to being exposed to parental behaviors. Although preliminary results, if supported by future studies, parental reports of their dispositional empathy could be identified in clinical screenings and used to target at-risk children. Moreover, parent-implemented communication interventions could also help parents and children to use communication, including emotional mimicry, without developing behavioral problems.

### 4.5. Limitations and Future Suggestions

Findings of the current study must be seen in light of the following limitations. First, the cross-sectional nature of the study precludes us from drawing specific conclusions about the developmental trajectories of infant emotional mimicry in triadic contexts of interaction. In order to provide more robust evidence on the specific contribution of parent dispositions and socialization experiences on emotional mimicry in the first year of life, longitudinal designs are necessary. Repeated observations over time would be important also to explore which developmental pathways are undertaken by infant emotional mimicry of positive and negative emotional stimuli (e.g., at what stage in development measures of positive and negative emotional mimicry become a more unitary construct, what developmental outcomes they relate to, and whether they convey distinct socially adaptive functions).

Second, only one of the parents participated in the study. Prior evidence indicates differences in mother-infant and father-infant interactive dynamics (for a review, see [114]). For example, during face-to-face interactions infants share more emotionality, in terms of smiles and gaze, with their mothers than with their fathers [11]. Our exploratory analyses performed by controlling for the effects of parental gender in the path models pointed out that infants share longer mutual attention with their mothers, as compared to fathers, also during triadic contexts of interaction. In our sample, mothers tended to mimic the fearful stimuli longer than fathers as well. Further, in line with prior research comparing women and men [58], our results indicated that maternal levels of affective empathy were significantly higher than paternal levels. Future research would therefore benefit from including in the research design both parents, investigating simultaneously the contribution of both maternal and paternal emotional and attentional responses on the infant emotional mimicry.

Third, the present study proposed an investigation of infant emotional mimicry in well-controlled experimental settings. Although we employed dynamic videos in which the emotional facial expression naturally unfolded (vs. static pictures), fewer than 50% of infants and parents displayed valence-congruent responses to the negative emotional stimuli. This might due to the fact that passively looking at a computer screen entails different motivations and dynamics than participating in social interactions in real life. In order to better examine the moderate level of responding, future studies should implement paradigms that involve social engagement with the model.

Last but not least, psychological research shows that spontaneous mimicry is sensitive to social cues (for a review, see [115]). Infants’ propensity to mimic might be modulated by the broader social contexts, including both individual states and traits (for a review, see [116]). Future research should, therefore, further investigate the contribution of the infant’s personal characteristics, as temperamental traits, in creating individual differences in infant emotional mimicry. Temperamental surgency and negative affectivity predispose the infant responses to the social environment in positive or negative ways. In addition to temperamental traits, the quality of the parent-child social interactions might condition infant responses to the emotional stimuli.

## 5. Conclusions

Emotional mimicry represents one of the earliest forms of human communication and provides implicit information about how to navigate the social world, especially for preverbal infants whose social interactions rely on interpersonal coordination of expressive behaviors. The findings of this study present new insights into infants’ and parents’ coordination of emotional mimicry and visual attention during triadic contexts of interactions, endorsing the social-affiliative function of mimicry present already in infancy. In fact, only when the observed emotional display is positive, and thus perceived as affiliative, emotional mimicry occurs as an automatic and spontaneous parent-infant shared behavior. Identifying which factors underpin individual differences in emotional mimicry as early as in the first year of life is the next step toward a better understanding of communicative development.

## Figures and Tables

**Table 1:** Descriptive statistics of the experimental variables (*N*_6-month_ = 51; *N*_12-month_ = 66; *N*_total_ = 117).

Experimental Variables	M (SE)			Range		
	6-Month	12-Month	Total	6-Month	12-Month	Total
Infant Emotional Mimicry						
Happy stimuli	15.57 (2.73)	16.77 (2.31)	16.24 (1.76)	0–68.15	0–65.98	0–68.15
Sad stimuli	5.38 (1.66)	5.32 (1.60)	5.35 (1.15)	0–50.00	0–54.00	0–54.00
Angry stimuli	5.21 (1.66)	5.45 (1.53)	5.34 (1.12)	0–50.00	0–51.00	0–51.00
Fearful stimuli	3.99 (1.51)	6.60 (1.55)	5.46 (1.09)	0–49.00	0–47.00	0–49.00
Parent Emotional Mimicry						
Happy stimuli	39.01 (3.54)	32.43 (3.07)	35.30 (2.33)	0–94.22	0–87.35	0–94.22
Sad stimuli	8.85 (1.92)	7.57 (1.57)	8.13 (1.22)	0–44.66	0–51.00	0–51.00
Angry stimuli	6.86 (1.62)	7.64 (1.54)	7.30 (1.12)	0–44.00	0–45.00	0–45.00
Fearful stimuli	2.08 (0.76)	4.11 (1.04)	3.22 (0.68)	0–29.04	0–32.00	0–32.00
Parent-Infant Attention						
Happy stimuli	2.90 (0.66)	3.17 (0.53)	3.05 (0.41)	0–21.00	0–19.85	0–21.00
Sad stimuli	2.90 (0.68)	3.21 (0.64)	3.07 (0.47)	0–20.00	0–19.00	0–20.00
Angry stimuli	2.20 (0.62)	3.61 (0.57)	2.99 (0.42)	0–18.00	0–19.00	0–19.00
Fearful stimuli	2.07 (0.47)	2.36 (0.40)	2.23 (0.30)	0–12.62	0–14.00	0–14.00
Parent Affective Empathy	3.97 (0.10)	4.00 (0.11)	3.99 (0.08)	2.14–5.29	1.86–5.71	1.86–5.71

**Table 2:** Correlations within the experimental constructs of infant and parent emotional mimicry, and parent-infant mutual attention.

Experimental Variables	1.	2.	3.	4.
Emotional Mimicry				
1. Happy stimuli	−	−0.16	−0.22 *	−0.19 *
2. Sad stimuli	0.36 **	−	0.83 *	0.66 **
3. Angry stimuli	0.40 **	0.50 **	−	0.73 **
4. Fearful stimuli	0.16	0.33 **	0.40 **	−
Parent-Infant Mutual Attention				
1. Happy stimuli	−			
2. Sad stimuli	0.47 **	-		
3. Angry stimuli	0.24 **	0.43 **	-	
4. Fearful stimuli	0.24 **	0.27 **	0.22 *	−

Note. * *p* < 0.05; ** *p* < 0.01. Infant and parent emotional mimicry are displayed in the top and bottom diagonals, respectively.

## Data Availability

The data presented in this study are openly available in FigShare at https://doi.org/10.6084/m9.figshare.13570562.v1.

## References

[1] Dufy K.A., Chartrand T.L. (2015). Mimicry: Causes and consequences. Curr. Opin. Behav. Sci..

[2] Hess U., Bourgeois P. (2010). You smile–I smile: Emotion expression in social interaction. Biol. Psychol..

[3] Sun X., Nijholt A. (2011). Multimodal embodied mimicry in interaction. Analysis of Verbal and Nonverbal Communication and Enactment. The Processing Issues.

[4] Lakin J.L., Chartrand T.L. (2003). Using nonconscious behavioral mimicry to create affiliation and rapport. Psychol. Sci..

[5] van Baaren R., Janssen L., Chartrand T.L., Dijksterhuis A. (2009). Where is the love? The social aspects of mimicry. Philos. Trans R. Soc. B.

[6] Chartrand T.L., Bargh J.A. (1999). The chameleon effect: The perception–behavior link and social interaction. J. Personal. Soc. Psychol..

[7] Over H., Carpenter M. (2012). Putting the social into social learning: Explaining both selectivity and fidelity in children’s copying behavior. J. Comp. Psychol..

[8] Lakin J.L., Chartrand T.L., Arkin R.M. (2008). I am too just like you: Nonconscious mimicry as an automatic behavioral response to social exclusion. Psychol. Sci..

[9] Datyner A.C., Richmond J.L., Henry J.D. The development of empathy in infancy: Insights from the rapid facial mimicry response. Proceedings of the ACNS-2013 Australasian Cognitive Neuroscience Society Conference.

[10] Decety J., Svetlova M. (2012). Putting together phylogenetic and ontogenetic perspectives on empathy. Dev. Cogn. Neurosci..

[11] Colonnesi C., Zijlstra B.J.H., van der Zande A., Bögels S.M. (2012). Coordination of gaze, facial expressions and vocalizations of early infant communication with mother and father. Infant Behav. Dev..

[12] de Klerk C.C., Lamy-Yang I., Southgate V. (2019). The role of sensorimotor experience in the development of mimicry in infancy. Dev. Sci..

[13] Soussignan R., Dollion N., Schaal B., Durand K., Reissland N., Baudouin J. (2018). Mimicking emotions: How 3–12-month-old infants use the facial expressions and eyes of a model. Cogn. Emot..

[14] Messinger D.S. (2002). Positive and negative: Infant facial expressions and emotions. Curr. Dir. Psychol. Sci..

[15] Grossmann T., Striano T., Friederici A.D. (2007). Developmental changes in infants’ processing of happy and angry facial expressions: A neurobehavioral study. Brain Cogn..

[16] Gwiazda J., Bauer J., Held R. (1989). From visual acuity to hyperacuity: A 10-year update. Can. J. Psychol..

[17] Ross P., Atkinson A.P. (2020). Expanding simulation models of emotional understanding: The case for different modalities, body-state simulation prominence, and developmental trajectories. Front. Psychol..

[18] Grossmann T. (2010). The development of emotion perception in face and voice during infancy. Restor. Neurol. Neurosci..

[19] Hoehl S. (2014). Emotion processing in infancy. Children and Emotion.

[20] Leppänen J.M., Nelson C.A. (2009). Tuning the developing brain to social signals of emotions. Nat. Rev. sNeurosci..

[21] Sorce J.F., Emde R.N., Campos J.J., Klinnert M.D. (1985). Maternal emotional signaling: Its effect on the visual cliff behavior of 1-year-olds. Dev. Psychol..

[22] Meltzoff A.N., Moore M.K. (1977). Imitation of facial and manual gestures by human neonates. Science.

[23] Meltzoff A.N., Murray L., Simpson E., Heimann M., Nagy E., Nadel J., Pedersen E.J., Brooks R., Messinger D.S., De Pascalis L. (2018). Re-examination of Oostenbroek et al. (2016): Evidence for neonatal imitation of tongue protrusion. Dev. Sci..

[24] Oostenbroek J., Suddendorf T., Nielsen M., Redshaw J., Kennedy-Costantini S., Davis J., Clark S., Slaughter V. (2016). Comprehensive longitudinal study challenges the existence of neonatal imitation in humans. Curr. Biol..

[25] Van der Donck S. (2020). Facing Emotions. Towards a Better Understanding of Automatic Facial Expression Processing Mechanisms in Typical and Atypical Populations. Ph.D. Thesis.

[26] Isomura T., Nakano T. (2016). Automatic facial mimicry in response to dynamic emotional stimuli in five-month-old infants. Proc. R. Soc. B.

[27] Kaiser J., Crespo-Llado M.M., Turati C., Geangu E. (2017). The development of spontaneous facial responses to others’ emotions in infancy: An EMG study. Sci. Rep..

[28] Flom R., Bahrick L.E. (2007). The development of infant discrimination of affect in multimodal and unimodal stimulation: The role of intersensory redundancy. Dev. Psychol..

[29] Datyner A., Henry J.D., Richmond J.L. (2017). Rapid facial reactions in response to happy and angry expressions in 7-month-old infants. Dev. Psychol..

[30] Bourgeois P., Hess U. (2008). The impact of social context on mimicry. Biol. Psychol..

[31] Hess U., Fischer A. (2013). Emotional mimicry as social regulation. Personal. Soc. Psychol. Rev..

[32] Kulesza W., Dolinski D., Szczęsna K., Kosim M., Grzyb T. (2019). Temporal Aspects of the Chameleon Effect and Hospitality: The Link Between Mimicry, Its Impact, and Duration. Cornell Hosp. Q..

[33] Zhou Y., Fischer M.H. (2018). Mimicking non-verbal emotional expressions and empathy development in simulated consultations: An experimental feasibility study. Patient Educ. Couns..

[34] Bandura A. (1977). Social Learning Theory.

[35] Jones S.S. (2009). The development of imitation in infancy. Philos. Trans. R. Soc. B.

[36] Malatesta C.Z., Haviland J.M. (1982). Learning display rules: The socialization of emotion expression in infancy. Child. Dev..

[37] Rayson H., Bonaiuto J.J., Ferrari P.F., Murray L. (2017). Early maternal mirroring predicts infant motor system activation during facial expression observation. Sci. Rep..

[38] Fogel A., Nwokah E., Dedo J.Y., Messinger D.S., Dickson K.L., Matusov E., Holt S.A. (1992). Social process theory of emotion: A dynamic systems approach. Soc. Dev..

[39] Jonsson C.O., Clinton D., Fahrman M., Mazzaglia G., Novak S., Sörhus K. (2001). How do mothers signal shared feeling—States to their infants? An investigation of affect attunement and imitation during the first year of life. Scand. J. Psychol..

[40] Nadel J., Meltzoff A.N., Prinz W. (2002). Imitation and imitation recognition: Functional use in preverbal infants and nonverbal children with autism. The Imitative Mind: Development, Evolution, and Brain Bases.

[41] Pawlby S., Schaffer H.R. (1977). Imitative interaction. Studies in Mother-Infant Interaction.

[42] Užgiris I.Č., Benson J.B., Kruper J.C., Vasek M.E. (1989). Contextual influences on imitative interactions between mothers and infants. Action in Social Context.

[43] Jones S.S. Infants learn to imitate by being imitated. Proceedings of the International Conference on Development and Learning: The Tenth International Conference on Development and Learning.

[44] Sauciuc G.A., Zlakowska J., Persson T., Lenninger S., Alenkaer Madsen E. (2020). Imitation recognition and its prosocial effects in 6-month old infants. PLoS ONE.

[45] Dimberg U., Andréasson P., Thunberg M. (2011). Emotional empathy and facial reactions to facial expressions. J. Psychophysiol..

[46] Sonnby–Borgström M. (2002). Automatic mimicry reactions as related to differences in emotional empathy. Scand. J. Psychol..

[47] Davis M.H. (2018). Empathy: A Social Psychological Approach.

[48] Eisenberg N., Fabes R.A., Miller P.A., Fultz J., Shell R., Mathy R.M., Reno R.R. (1989). Relation of sympathy and personal distress to prosocial behavior: A multimethod study. J. Personal. Soc. Psychol..

[49] Volling B.L., Kolak A.M., Kennedy D.E., Fehr B., Sprecher S., Underwood L.G. (2008). Empathy and compassionate love in early childhood: Development and family influence. The Science of Compassionate Love: Theory, Research, and Applications.

[50] Emery N.J. (2000). The eyes have it: The neuroethology, function and evolution of social gaze. Neurosci. Biobehav. Rev..

[51] Prochazkova E., Kret M.E. (2017). Connecting minds and sharing emotions through mimicry: A neurocognitive model of emotional contagion. Neurosci. Biobehav. Rev..

[52] Campos J.J. (1983). The importance of affective communication in social referencing: A commentary on Feinman. Merrill-Palmer Q..

[53] Zeegers M.A., Potharst E.S., Veringa-Skiba I.K., Aktar E., Goris M., Bögels S.M., Colonnesi C. (2019). Evaluating mindful with your baby/toddler: Observational changes in maternal sensitivity, acceptance, mind-mindedness, and dyadic synchrony. Front. Psychol..

[54] de Klerk C.C., Hamilton A.F.D.C., Southgate V. (2018). Eye contact modulates facial mimicry in 4-month-old infants: An EMG and fNIRS study. Cortex.

[55] Wang Y., Hamilton A.F.D.C. (2014). Why does gaze enhance mimicry? Placing gaze-mimicry effects in relation to other gaze phenomena. Q. J. Exp. Psychol..

[56] Kotsoni E., de Haan M., Johnson M.H. (2001). Categorical perception of facial expressions by 7-month-old infants. Perception.

[57] Adamson L.B. (1995). Communication Development during Infancy.

[58] Davis M.H. (1980). A multidimensional approach to individual differences in empathy. JSAS Cat. Sel. Doc. Psychol..

[59] De Corte K., Buysse A., Verhofstadt L.L., Roeyers H., Ponnet K., Davis M.H. (2007). Measuring empathic tendencies: Reliability and validity of the Dutch version of the Interpersonal Reactivity Index. Psychol. Belg..

[60] van der Schalk J., Hawk S.T., Fischer A.H., Doosje B. (2011). Moving faces, looking places: Validation of the Amsterdam Dynamic Facial Expression Set (ADFES). Emotion.

[61] Ekman P., Friesen W.V. (1978). The Facial Action Coding System (FACS).

[62] Noldus L.P., Trienes R.J., Hendriksen A.H., Jansen H., Jansen R.G. (2000). The Observer Video-Pro: New software for the collection, management, and presentation of time-structured data from videotapes and digital media files. Behav. Res. Methods Instrum. Comput..

[63] Graham J.W. (2009). Missing data analysis: Making it work in the real world. Annu. Rev. Psychol..

[64] Tabachnick B.G., Fidell L.S. (2013). Using Multivariate Statistics.

[65] Rosseel Y. (2012). Lavaan: An R package for structural equation modeling. J. Stat. Softw..

[66] Horstmann G. (2003). What do facial expressions convey: Feeling states, behavioral intentions, or action requests?. Emotion.

[67] Yik M.S. (1999). Interpretation of faces: A cross-cultural study of a prediction from Fridlund’s theory. Cogn. Emot..

[68] Hendriks M.C., Vingerhoets A.J. (2006). Social messages of crying faces: Their influence on anticipated person perception, emotions and behavioural responses. Cogn. Emot..

[69] Olderbak S., Sassenrath C., Keller J., Wilhelm O. (2014). An emotion-differentiated perspective on empathy with the emotion specific empathy questionnaire. Front. Psychol..

[70] Bigelow A.E., Power M., Bulmer M., Gerrior K. (2015). The relation between mothers’ mirroring of infants’ behavior and maternal mind-mindedness. Infancy.

[71] Aktar E., Bögels S.M. (2017). Exposure to parents’ negative emotions as a developmental pathway to the family aggregation of depression and anxiety in the first year of life. Clin. Child Fam. Psychol. Rev..

[72] Aktar E., Pérez-Edgar K., Lockman J., Tamis-LeMonda C. (2020). Infant Emotion Development and Temperament. The Cambridge Handbook of Infant Development: Brain, Behavior, and Cultural Context.

[73] Ray E., Heyes C. (2011). Imitation in infancy: The wealth of the stimulus. Dev. Sci..

[74] Aktar E., Colonnesi C., de Vente W., Majdandžić M., Bögels S.M. (2017). How do parents’ depression and anxiety, and infants’ negative temperament relate to parent–infant face-to-face interactions?. Dev. Psychopathol..

[75] Aktar E., Majdanžić M., De Vente W., Bögels S.M. (2013). The interplay between expressed parental anxiety and infant behavioural inhibition predicts infant avoidance in a social referencing paradigm. J. Child Psychol. Psychiatry.

[76] De Rosnay M., Cooper P.J., Tsigaras N., Murray L. (2006). Transmission of social anxiety from mother to infant: An experimental study using a social referencing paradigm. Behav. Res. Ther..

[77] Skinner A.L., Meltzoff A.N., Olson K.R. (2017). Catching social bias: Exposure to biased nonverbal signals creates social biases in preschool children. Psychol. Sci..

[78] Bandura A., Rosenthal T.L. (1996). Vicarious classical conditioning as a function of arousal level. J. Personal. Soc. Psychol..

[79] Hess U., Fischer A.H. (2016). Emotional Mimicry in Social Context.

[80] Eisenberg N., Fabes R.A., Schaller M., Carlo G., Miller P.A. (1991). The relations of parental characteristics and practices to children’s vicarious emotional responding. Child Dev..

[81] Davis M.H., Luce C., Kraus S.J. (1994). The heritability of characteristics associated with dispositional empathy. J. Personal..

[82] McDonald N.M., Messinger D.S. (2011). The development of empathy: How, when, and why. Moral Behav. Free Will A Neurobiol. Philos. Aprroach.

[83] Schwarz N., Clore G.L., Kruglanski A., Higgins E.T. (1996). Feelings and phenomenal experiences. Social Psychology: Handbook of Basic Principles.

[84] Lepaennen J.M., Hietanen J.K. (2004). Positive facial expressions are recognized faster than negative facial expressions, but why?. Psychol. Res..

[85] Yabar Y., Cheung N., Hess U., Rochon G., Bonneville-Hébert N. Dis-moi si vous êtes intimes, et je te dirais si tu mimes. [Tell me if you’re intimate and I’ll tell you if you’ll mimic]. Proceedings of the 24th Annual Meeting of the Société Québécoise pour la Recherche en Psychologie.

[86] Manstead A.S.R., Fischer A.H., Scherer K.R., Schorr A., Johnstone T. (2001). Social appraisal: The social world as object of and influence on appraisal processes. Appraisal Processes in Emotion: Theory, Methods, Research.

[87] Kiley Hamlin J., Wynn K., Bloom P. (2010). Three-month-olds show a negativity bias in their social evaluations. Dev. Sci..

[88] Vaish A., Grossmann T., Woodward A. (2008). Not all emotions are created equal: The negativity bias in social-emotional development. Psychol. Bull..

[89] Jessen S., Altvater-Mackensen N., Grossmann T. (2016). Pupillary responses reveal infants’ discrimination of facial emotions independent of conscious perception. Cognition.

[90] Addabbo M., Vacaru S.V., Meyer M., Hunnius S. (2020). ‘Something in the way you move’: Infants are sensitive to emotions conveyed in action kinematics. Dev. Sci..

[91] Sroufe L.A. (1995). Emotional Development: The Organization of Emotional Life in the Early Years.

[92] Fischer A.H., Dukes D., Clément F. (2019). Learning from others’ emotions. Foundations of Affective Social Learning: Conceptualizing the Social Transmission of Value.

[93] Mann L., Feddes A.R., Doosje B., Fischer A.H. (2016). Withdraw or affiliate? The role of humiliation during initiation rituals. Cogn. Emot..

[94] Wheeler L., Suls J.A., Suls J., Collins R.L., Wheeler L. (2020). History of Social Comparison Theory. Social Comparison, Judgment, and Behavior.

[95] Lee V. (2016). Examining the Perception of Emotional Facial Expressions in Early Childhood. Ph.D. Thesis.

[96] Taipale J. (2016). Self-regulation and beyond: Affect regulation and the infant–caregiver dyad. Front. Psychol..

[97] Barrett K.C., Campos J.J., Osofsky J.D. (1987). Perspectives on emotional development II: A functionalist approach to emotions. Handbook of Infant Development.

[98] Izard C.E. (1991). Emotions, Personality, and Psychotherapy. The Psychology of Emotions.

[99] Vuilleumier P. (2002). Facial expression and selective attention. Curr. Opin. Psychiatry.

[100] Fischer A.H., Manstead A.S., Lewis M., Haviland-Jones J., Barrett L.F. (2008). Social functions of emotion. Handbook of Emotions.

[101] Hess U., Fischer A. (2014). Emotional mimicry: Why and when we mimic emotions. Soc. Personal. Psychol. Compass.

[102] Southgate V., Hamilton A.F.D.C. (2008). Unbroken mirrors: Challenging a theory of autism. Trends Cogn. Sci..

[103] Oberman L.M., Winkielman P., Ramachandran V.S. (2009). Slow echo: Facial EMG evidence for the delay of spontaneous, but not voluntary, emotional mimicry in children with autism spectrum disorders. Dev. Sci..

[104] Vivanti G., Hamilton A., Volkmar F., Paul R., Rogers S., Pelphrey K. (2014). Imitation in autism spectrum disorders. Handbook of Autism and Pervasive Developmental Disorders.

[105] American Psychiatric Association (APA) (2013). Diagnostic and Statistical Manual of Mental Disorders: DSM-V.

[106] Dawson G. (2008). Early behavioral intervention, brain plasticity, and the prevention of autism spectrum disorder. Dev. Psychopathol..

[107] Chawarska K., Macari S., Shic F. (2013). Decreased spontaneous attention to social scenes in 6-month-old infants later diagnosed with autism spectrum disorders. Biol. Psychiatry.

[108] Ozonoff S., Iosif A.M., Baguio F., Cook I.C., Hill M.M., Hutman T., Rogers S.J., Rozga A., Sangha S., Sigman M. (2010). A prospective study of the emergence of early behavioral signs of autism. J. Am. Acad. Child Adolesc. Psychiatry.

[109] Wallace K.S., Rogers S.J. (2010). Intervening in infancy: Implications for autism spectrum disorders. J. Child Psychol. Psychiatry.

[110] Morris A.S., Silk J.S., Steinberg L., Myers S.S., Robinson L.R. (2007). The role of the family context in the development of emotion regulation. Soc. Dev..

[111] de Paul J., Perez-Albeniz A., Guibert M., Asla N., Ormaechea A. (2008). Dispositional empathy in neglectful mothers and mothers at high risk for child physical abuse. J. Interpers. Violence.

[112] Beardslee W.R., Gladstone T.R., O’Connor E.E. (2011). Transmission and prevention of mood disorders among children of affectively ill parents: A review. J. Am. Acad. ChildAdolesc. Psychiatry.

[113] Field T.M., Hernandez-Reif M., Vera Y., Gil K., Diego M., Bendell D., Yando R. (2005). Anxiety and anger effects on depressed mother–infant spontaneous and imitative interactions. Infant Behav. Dev..

[114] Möller E.L., Majdandžić M., De Vente W., Bögels S.M. (2013). The evolutionary basis of sex differences in parenting and its relationship with child anxiety in Western societies. J. Exp. Psychopathol..

[115] Palagi E., Celeghin A., Tamietto M., Winkielman P., Norscia I. (2020). The neuroethology of spontaneous mimicry and emotional contagion in human and non-human animals. Neurosci. Biobehav. Rev..

[116] Seibt B., Mühlberger A., Likowski K., Weyers P. (2015). Facial mimicry in its social setting. Front. Psychol..

